# The serial mediating effects of learning motivation and learning engagement on the relationship between gen AI literacy and EFL perceived proficiency among vocational students

**DOI:** 10.3389/fpsyg.2026.1862619

**Published:** 2026-07-22

**Authors:** Rui Zhang, Othman Bin Talib, Shengnan Liu, Zhixinlu Chen, Daliang Lu, Yufang Meng

**Affiliations:** 1 Faculty of Social Science and Liberal Arts of UCSI, UCSI University, Kuala Lumpur, Malaysia; 2 Department of Information and Engineering, Liaoning Mechatronics College, Dandong City, China

**Keywords:** EFL perceived proficiency, generative artificial intelligence, learning engagement, learning motivation, self-determination theory

## Abstract

Generative artificial intelligence (Gen AI) has emerged as a transformative technology in English language education. However, research examining the serial mediating roles of learning motivation and learning engagement in the relationship between Gen AI and vocational students’ English as a Foreign Language (EFL) proficiency remains scarce. This study employed a cross-sectional survey design involving 383 vocational college students in China and utilized validated instruments measuring Gen AI literacy, learning motivation, learning engagement, and EFL perceived proficiency. The results indicated that Gen AI exerted a significant positive direct effect on EFL perceived proficiency and a strong positive effect on learning motivation. However, its direct effect on learning engagement was not statistically significant. Learning motivation significantly predicted learning engagement, whereas learning engagement was a strong predictor of EFL perceived proficiency. Furthermore, the serial mediating effect of learning motivation and learning engagement was significant, whereas their individual mediating effects were not statistically significant. These findings extend Self-Determination Theory (SDT) by demonstrating that the influence of Gen AI on EFL perceived proficiency operates primarily through a motivation–engagement pathway rather than through direct technological exposure. The results also provide practical implications for the design of engagement-oriented, AI-supported language learning environments in vocational education.

## 1 Introduction

In contemporary educational contexts, English proficiency is increasingly recognized as a critical competency for the academic and career development of vocational students (Li et al., 2025; Peng and Wang, 2025). Although the impact of generative artificial intelligence (Gen AI) on university students’ English as a Foreign Language (EFL) proficiency has been extensively examined in recent reviews and empirical studies (Chan et al., 2024; Wang et al., 2024), vocational college students remain a largely underexplored population (Cao and Abdullah, 2025). According to a survey conducted by the UNESCO International Institute for Educational Planning, nearly one-quarter of existing occupations are expected to undergo substantial technological transformation, highlighting the urgent need for vocational education and training systems to adapt to emerging labor-market demands (International Institute for Educational Planning, 2025). Similarly, a national survey conducted by the Center for Vocational Education Development of the Chinese Ministry of Education revealed that although 99.4% of vocational students reported using AI-related learning resources, more than 60% lacked systematic practical training (Fang et al., 2026). These findings underscore the importance of investigating how Gen AI can effectively support vocational students’ language learning and professional development.

In contrast to earlier artificial intelligence systems that predominantly depend on predictive models derived from historical datasets. Gen AI is capable of delivering prompt and meaningful feedback essential for language development and providing tailored recommendations (Law, 2024; Noroozi et al., 2024). In the literature for the past 5 years, there have been various applications of Gen AI, including generating realistic images and videos that help students with their learning (Bakpayev et al., 2020; Elasri et al., 2022) Fostering Gen AI in English education is necessary in that a significant body of literature indicates that AI could deliver customized instructional materials (Estaiteyeh and McQuirter, 2024), and facilitate intelligent teaching practices (Chu, 2024), provide students with timely feedback (Urban et al., 2024). However, there are challenges posed by the limitations of GenAI, such as encouraging superficial study and relying on automated support excessively (Kasneci et al., 2023), which compromise students’ genuine efforts to develop their language-learning competence.

Empirical evidence regarding whether Gen AI directly improves motivation in the EFL context remains inconsistent, especially among vocational students. Some studies focus primarily on the impact of AI on learning outcomes (Huang et al., 2021; Zeng et al., 2020), while others discuss mediating factors such as learning motivation and engagement among university students (Shloul et al., 2024; Zhao et al., 2023). In a report released by Modern Higher Vocational Colleges Technical network (Zeng et al., 2020) which is related to China’s National Association of Vocational College Presidents, 101,233 vocational students from 203 higher vocational schools, 44.3% of vocational students reported a lack of learning motivation, which mainly comes from employment, compared with university students’ motivation of enrolling in a higher-level school and going abroad. These motivational differences suggest a need to investigate the motivating factors shaping vocational students’ language study (Wu, 2024).

Learning motivation, however, may not directly translate into improved language proficiency unless it is accompanied by active learning engagement. Engagement is a strong predictor of success in EFL contexts (Hiver et al., 2021). Due to limited teaching hours and a lack of effective learning methods, nearly 50% of vocational students have not taken Gen AI courses (Fang et al., 2026), many of whom have low engagement with English instruction (Muliyah and Aminatun, 2020). Nevertheless, limited research has examined whether learning motivation and learning engagement jointly explain how Gen AI influences English language learning outcomes. And the vocational education settings are often neglected (Ruijuan et al., 2023).

Although previous studies have established positive associations between AI-supported learning and language-learning outcomes, several important gaps remain. First, most existing studies focus on university students, while vocational college students remain underrepresented. Second, previous research has primarily examined direct relationships between Gen AI use and learning outcomes, providing limited insight into the psychological mechanisms underlying these relationships. Third, little empirical evidence is available regarding the serial process through which Gen AI may influence English proficiency via learning motivation and learning engagement. Addressing these gaps can contribute to a more comprehensive understanding of how AI-supported learning environments facilitate language development.

To address these limitations, the present study draws upon Self-Determination Theory (SDT) (Ryan and Deci, 2017) to investigate the relationships among Gen AI, learning motivation, learning engagement, and EFL proficiency among Chinese vocational college students. Specifically, this study proposes and tests a serial mediation model between Gen AI and EFL perceived proficiency. By focusing on vocational students and examining both motivational and engagement pathways, this study contributes to literature in three important ways. First, it extends existing research on AI-assisted language learning by examining an underrepresented student population. Second, it enriches the application of SDT in AI-supported educational contexts. Third, it provides practical implications for educators to design effective, engagement-oriented AI-supported language learning environments in vocational education.

Therefore, there is a need to address the critical research gap. This study provides two novel contributions. Theoretically, it advances current understandings of Gen AI through SDT. Practically, prior studies predominantly examine motivation and engagement independently among university students, while this research identifies serial motivational and engagement factors that can be leveraged to enhance vocational EFL perceived proficiency.

## 2 Literature review

### 2.1 Generative artificial intelligence in EFL

Feuerriegel et al. (2023) defined Gen AI as a form of generative modeling implemented through machine learning architectures capable of producing new data samples based on learned patterns. It represented a transformative advancement in educational technology, fundamentally distinct from earlier digital tools and capable of reshaping traditional teaching and learning models (Swindell et al., 2024). Zhou et al. (2024) gave the operational definition Gen AI as comprehensive and multidimensional analysis of encompassing AI knowledge levels, skills in using AI products, attitudes toward use of Gen AI, and ethics in Gen AI usage. In a report on a large-scale multi-institution survey of over 8,000 students studying at four Australian universities, more than 80% percent of students used Gen AI such as Gemini, Microsoft Copilot and Claude to brainstorm ideas and to learn vocabulary, with nearly half using it regularly to summarise texts, grammar checking, draft assignments and edit writing (Henderson et al., 2026). It had been widely discussed in terms of its strengths, weaknesses, opportunities, and threats in education since its release in 2022 (Lo et al., 2024).

In this study, foreign language ability referred to English proficiency acquired after the mastery of one’s native language (Ellis, 1994). It was essential for educators to integrate Gen AI into their study for professional development (Kong, 2024) as AI was associated with enhanced performance in listening, speaking, reading, and writing skills, ultimately contributing to students’ overall academic success (Wu and Kang, 2023). English proficiency was assessed across four dimensions: listening, speaking, reading, and writing (Ruegg et al., 2024).

In empirical studies, GenAI tools are also believed to be useful research aids for generating ideas, synthesizing information, and summarising a vast amount of text data to help researchers analyse data and compose their writing (Berg, 2023). GenAI can also bring benefits in learning assessment (Crompton and Burke, 2023). Mizumoto and Eguchi (2023) examined the reliability and accuracy of ChatGPT as an automated essay scoring tool, which was able to provide immediate scores and feedback on students’ writing skills. Such research demonstrates that GenAI has the potential to transform the teaching and learning process as well as improve student learning outcomes. However, the use of Gen AI also faced many problems, which remain unaddressed by the existing research (Fang et al., 2026) in higher vocational colleges such as lack of sufficient cognitive effort and laziness (Kasneci et al., 2023), thereby supporting the formulation of the first hypothesis, H1.

*H1*: Gen AI is positively related to vocational students’ EFL perceived proficiency.

### 2.2 Learning motivation

Learning motivation refers to the willingness or desire to engage in activities that drive goal-directed behavior, effort, and persistence (Hacker, 1976). It is a motivational tendency that initiates and sustains academic behavior, directing it toward specific academic goals (Liu, 2025), which can enhance learning engagement and improve academic performance (Liu et al., 2024a,b).

Learning motivation can be broadly categorized into three forms: intrinsic motivation, extrinsic motivation, and amotivation (Ryan and Deci, 2020). They influenced learners’ language learning behavior through distinct mechanisms (Zheng, 2021), in which intrinsic motivation has been found to exert a significantly greater mediation effect than extrinsic motivation (Peng and Fu, 2021). In theories relating to factors influencing motivation, SDT remained the most appropriate framework for examining psychological need satisfaction in AI-supported language learning (Ntoumanis et al., 2020). Within the SDT framework, some research showed that the use of AI tools can significantly enhance motivation in the language learning process (Noroozi et al., 2024; Xiao et al., 2024).

In the last 5 years, Nozhovnik et al. (2023) highlighted the positive effects of gamified chatbots on undergraduates’ English language skills and motivation in second-language e-classes. Lee and Yeo (2022) confirmed that using AI-based chatbots for after-school review can significantly improve students’ motivation to learn and academic performance in public health courses. Huang and Mizumoto (2024) specifically examined the impact of ChatGPT on student motivation in EFL learning and found that students who utilized ChatGPT reported higher levels of motivation. These findings all hint at the great potential of Gen AI to boost university students’ motivation in learning, but there are few studies on Gen AI playing a positive role on vocational students’ motivation, which leads to the second hypothesis, H2.

*H2*: Gen AI is positively related to students’ learning motivation.

What’s more, enhanced intrinsic motivation promoted better performance and well-being among students (Howard et al., 2021). Motivated learners were more likely to tolerate communication anxiety, maintain long-term practice, and persist despite slow progress (Amoah and Yeboah, 2021). Research also showed that advanced learners often demonstrate stronger motivational orientations and increase learning effort in EFL perceived proficiency (Alsmari, 2023), thereby improving their academic performance (Ghavamnia and Kashkouli, 2022; Hellín et al., 2023), which may lead to the third hypothesis, H3.

*H3*: Learning motivation is positively associated with vocational students’ EFL perceived proficiency.

### 2.3 Learning engagement

Learning engagement is the investment of physical, cognitive, and emotional energy in learning (Kahu, 2011). Cognitive, behavioral, and emotional engagement are the three specific manifestations of learning engagement (Fredricks et al., 2004). Agentic engagement complements the traditional three dimensions as the fourth dimension of student engagement (Reeve and Tseng, 2011). Cognitive engagement focuses on students’ self-efficacy, learning goals, and self-regulation, reflecting the intellectual effort they put into their learning (Santos et al., 2023). Behavioral engagement refers to students’ observable participation in learning activities, including attendance, effort, persistence, attention, and active involvement in academic tasks (Skinner et al., 2008). Emotional engagement refers to the learners’ emotional response to school, learning, and the academic community, such as interest, enjoyment, and happiness (Fredricks et al., 2004). Agentic engagement describes students’ active efforts to enrich their own and others’ learning experiences (Reeve and Tseng, 2011). Learning engagement significantly predicts motivation, highlighting its role as a critical mechanism through which students achieve successful learning outcomes (Reeve et al., 2025).

Motivation has been identified as a critical predictor of learners’ engagement from SDT theory (Liu et al., 2026). However, excessively controlled motivation may hinder engagement by increasing anxiety, fear of making mistakes, and fear of negative evaluation, which can reduce learners’ engagement in communicative interactions (Amoah and Yeboah, 2021).

From the perspective of factors affecting engagement, Martin et al. (2020) concluded that there was a growing trend of studying student engagement in online learning studies. Research has shown that experience and attitude have a great influence on students’ engagement and disengagement (Bergdahl and Bond, 2021). However, seldom are vocational students’ learning motivation and engagement considered before.

From the perspective of related theory, within the SDT framework, student engagement is often conceptualized as a motivational outcome that emerges when students’ needs for autonomy, competence, and relatedness are satisfied (Ryan and Deci, 2020). Liu (2025) proposed that intrinsic motivation was one of the most important foundations necessary for academic development, which posed the fourth hypothesis, H4.

*H4*: Learning motivation is positively related to vocational students’ engagement.

Empirical evidence indicated that AI-driven personalized instruction can significantly increase classroom engagement (Yuan et al., 2025). Gjermeni and Prodani (2024) investigated in detail the impact of Gen AI on student engagement by using a comparative analysis of different AI-powered educational technologies. From the SDT perspective, Gen AI afforded greater learner autonomy and interactivity, leading to enhanced engagement (Guan et al., 2024; Liu et al., 2026). Aligning AI-supported learning with learners’ ideal self-concepts may enhance learning enjoyment, which can partially increase cognitive engagement (Liu et al., 2024a,b; Noroozi et al., 2024). Chan and Hu (2023) did a qualitative survey of 399 undergraduate and postgraduate students from various disciplines in Hong Kong, revealing a generally positive attitude towards GenAI in teaching and learning engagement.

More research is still needed on the effects of Gen AI on student academic engagement in literature classes and the corresponding emotional experiences (Guo and Wang, 2024). But given the ecologically situated and dynamic nature of engagement, there has been a growing call for research investigating this construction beyond isolated tasks (Hiver et al., 2021), As Cai et al. (2023) discovered, overdependence on Gen AI diminished intellectual engagement, potentially resulting in a decrease in critical thinking, with students making decisions solely based on the information provided by Gen AI (Chan and Hu, 2023). which leads to the fifth hypothesis, H5.

*H5*: Gen AI is positively related to vocational students’ engagement.

Research also indicated that foreign language enjoyment was strongly correlated with learner engagement (Guo, 2021; Dahri et al., 2025; Li and Chiu, 2025). Compared with traditional collaborative activities, Suciati et al. (2024) discovered that cognitive, behavioral, and emotional engagement based on Gen AI technology can enhance learners’ outcomes in EFL studies. However, some students have reported negative effects of Gen AI on their engagement with peers and teachers (Akpen et al., 2024) besides, vocational college students constitute a population that has received limited research attention (Cao and Abdullah, 2025), which forms the sixth hypothesis, H6.

*H6*: Engagement is positively related to vocational students’ EFL perceived proficiency.

### 2.4 The mediating effect of learning motivation and engagement on the relationship between gen AI and EFL perceived proficiency

From the empirical study of SDT, these innovative pedagogical strategies can satisfy learners’ psychological needs autonomously, impact student motivation and therefore boost academic achievement (Lee and Yeo, 2022). In contrast to traditional methods, the use of Gen AI tools in educational settings can increase students’ motivation and encourage them to engage more in learning activities (Hellín et al., 2023; Liu et al., 2024a,b). In this process, Gen AI may first enhance motivation, then transform it into active participation, cognitive investment and emotional involvement, and finally improve language proficiency (Li and Chiu, 2025). This serial process challenges techno-deterministic assumptions that technology directly produces learning outcomes and leads to the seventh hypothesis:

*H7*: Learning motivation mediates the relationship between Gen AI and EFL perceived proficiency.

Furthermore, the integration of Gen AI was also found to influence learner engagement, which may function as a mediating mechanism linking AI-assisted learning and improvements in English proficiency (Hellín et al., 2023). In this process, students may experience diverse emotions and affective states in technology-supported settings (Shao et al., 2023), which may influence their learning outcomes indirectly through behavioral engagement, highlighting the critical role of engagement in facilitating effective learning processes (Feng and Hong, 2022; Wang et al., 2023), and therefore results in the eighth hypothesis:

*H8*: Engagement mediates the relationship between Gen AI and EFL perceived proficiency.

Moreover, existing literature also provided substantial evidence regarding the role of Gen AI in shaping both motivation and engagement within English language learning contexts (Jamshed et al., 2024). From the perspective of SDT, the connection between motivation and engagement can be understood through the fulfillment of fundamental psychological needs, namely autonomy, competence, and relatedness, which fostered intrinsic motivation and subsequently encourage deeper engagement in learning activities (Ryan and Deci, 2000). From the perspective of empirical methods. Li et al. (2024) studied the relation between lerning motivation and engagement among university students. However, few studies have examined the serial mediating effect of learning motivation and engagement in a foreign language learning context among vocational students. Accordingly, the following hypotheses are proposed:

*H9*: Learning motivation and engagement serially mediate the relationship between Gen AI and EFL perceived proficiency.

### 2.5 Conceptual framework

The conceptual framework of this study is grounded in the principles of SDT. Different from Cognitive Theory, which posits that learners construct knowledge through the processes of perception, attention, encoding, storage, and retrieval of information (Schunk, 2020), SDT emphasizes that individuals are more likely to engage in learning activities when their psychological needs for autonomy, competence, and relatedness are satisfied. In the context of EFL learning, the emergence of Gen AI has created new opportunities for personalized, autonomous, and interactive language learning experiences. Accordingly, this study proposes a framework in which technology-enhanced language learning can achieve learning motivation, which subsequently promotes active behavioral engagement and leads to improved learning outcomes (Ryan and Deci, 2000, 2020).

**Figure 1:**
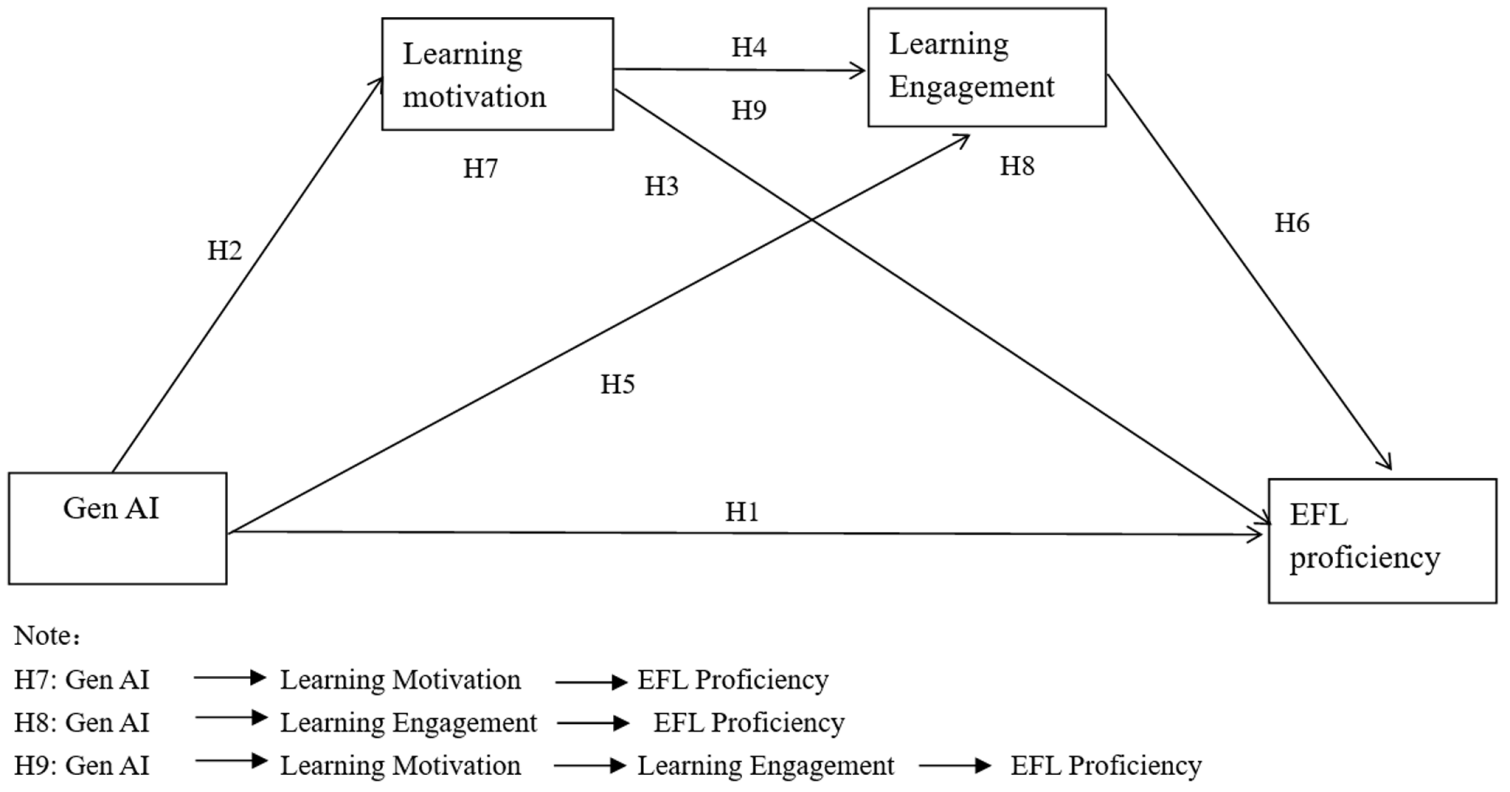
Architecture diagram of the research hypothesis.

This study establishes a hypothetical model based on the literature review and proposed research hypotheses. The model uses Gen AI as an independent variable, EFL as a dependent variable, and learning motivation and learning engagement as mediating variables. The proposed conceptual framework assumes that Gen AI may directly influence students’ EFL perceived proficiency while also indirectly affecting proficiency through the mediating roles of learning motivation and engagement, as shown in Figure 1.

## 3 Methodologies

### 3.1 Sample selection

Prior to data collection, permission was obtained from participating institutions. Students were informed about the purpose of the study, voluntary participation, anonymity, and confidentiality of their responses. Participation was entirely voluntary, and respondents were allowed to withdraw from the survey at any stage without penalty.

In PLS-SEM, the sample size should be equal to 10 times the number of independent variables in the most complex regression (Hair et al., 2017), which is at least 140 people in this study. According to Krejcie and Morgan (1970), and considering the large population of approximately 5.68 million vocational college students in China (Ministry of Education of China, 2021), a minimum sample size of 383 is deemed sufficient to achieve adequate statistical power. Therefore, the final valid sample of 383 respondents was considered statistically adequate and methodologically robust.

Participants were recruited from vocational colleges in Liaoning Province, China, who had prior experience using Gen AI tools for English learning where English is a compulsory component of the general curriculum. Data was collected from three vocational colleges in northeast China, representing different academic orientations-two science and engineering colleges and one liberal arts college. A convenience sampling strategy was employed to facilitate access to participants while ensuring diversity in disciplinary backgrounds. Questionnaires were collected online by Wenjuanxing between October and December 2025. Institutional permission was obtained before data collection, and participation was voluntary and anonymous. A total of 427 questionnaires were returned. After removing incomplete responses, duplicate submissions, and cases showing clear response patterns, 383 valid responses were retained, yielding an effective response rate of 89.7%.

### 3.2 Instruments

This study employed four validated questionnaires, SPSS 27.0, and Smart-PLS 4.0 for structural equation modelling for preliminary data analysis to comprehensively examine the interconnected effects of Gen AI, learning motivation, and engagement on vocational students’ EFL perceived proficiency. Some examples of detailed measurement items used in this study are presented in the Appendix. The questionnaire is divided into two parts. The first part is demographic information including gender, age and major. and the second part consists of four questionnaires with a 5-point Likert scale ranging from “Strongly Disagree (1)” to “Strongly Agree (5)”, the result of which is used in SPSS 27.0 and Smart PLS 4.0.

Gen AI Literacy questionnaire (Zhou et al., 2024) measures the extent to which learners actively participate in Gen AI learning, including Gen AI knowledge, Gen AI skills, Gen AI attitudes and Gen AI ethics. Questions include “I am able to select the appropriate AI to solve problems.” “I believe AI can provide me with unique insights and perspectives.” “I believe it is relatively easy to learn how to use new AI products.”

Language Learning Motivation Scale (Noels et al., 2000) allows for a nuanced analysis of how different types of motivation mediate the relationship between Gen AI and EFL speaking proficiency, which contains three dimensions of inner motivation, external motivation and amotivation with Cronbach’s *α* from 0.73 to 0.95 (Noels et al., 2000). Questions include “I am learning English because I enjoy the feeling of acquiring knowledge about the second language community and their way of life.” “In order to have a better salary later on.” “Honestly, I don’t know, I truly have the impression of wasting my time in studying a second language.”

Engagement in School-Four-Dimensional Scale (Veiga et al., 2014) is designed to capture engagement as a multidimensional construct, which reflects the extent to which learners actively participate in the learning process, which contains cognitive engagement, emotional engagement, behavior engagement and agentic engagement, with Cronbach’s α 0.82 and its different dimensions varying from 0.70 to 0.87 (Veiga et al., 2014). Questions include “When writing my work, I begin by making a plan for drafting the text.” “My school is a place where I make friends easily.” “I make suggestions to teachers about how to improve classes.” This questionnaire was also used for similar research (Ghavamnia and Kashkouli, 2022). However, the behavioral engagement dimension primarily reflected general school behavior and classroom discipline (e.g., “being absent without valid reason,” “intentionally disturbing the class”), which showed weak relevance to the context of AI-assisted foreign language learning. Considering structural validity and contextual appropriateness, these items were removed from the final scale.

Chinese perceived EFL perceived proficiency (Ministry of Education of the People's Republic of China, 2018) is a questionnaire adapted to measure the overall competence of English learners, including language proficiency such as listening, speaking, reading and writing, grammar proficiency and pragmatic proficiency. The questionnaire was modified according to the Chinese students’ vocational learning status. Questions include “I can understand the main content when listening to or watching radio and television programmes on general topics.” “I can write reports related to one’s own professional field, such as book reviews and research reports, with a complete structure.” “I am able to understand the views, emotions, attitudes and intentions expressed by others in common social situations.”

SmartPLS 4.0 was employed to test hypothesized relationships. PLS-SEM was selected for three reasons. First, the study was prediction-oriented and focused on explaining the serial mediating mechanisms linking Gen AI Literacy and EFL perceived proficiency rather than confirming an established covariance structure. Second, the proposed model involved multiple mediation paths and higher-order conceptual relationships, making PLS-SEM appropriate for handling complex structural models. Third, a preliminary normality assessment indicated deviations from multivariate normality, for which PLS-SEM is more robust than covariance-based SEM. Bootstrapping with 5,000 resamples was performed to estimate path coefficients, indirect effects, and significance levels. The analysis followed the standard two-step procedure: first assessing the measurement model and then evaluating the structural model.

### 3.3 Reliability and validity of the questionnaires

Reliability and convergent validity were assessed using Cronbach’s alpha, composite reliability (CR), and average variance extracted (AVE). As shown in Table 1, all Cronbach’s alpha values exceeded 0.70, all CR values were above the recommended threshold of 0.70, and all AVE values exceeded 0.50, indicating satisfactory internal consistency and convergent validity.

**Table 1:** Reliability and convergent validity.

Item	Outer loading range	Cronbach’s alpha	Composite reliability (rho_c)	Average variance extracted (AVE)
AI1-AI14	0.705–0.859	0.962	0.966	0.67
IM1-IM3	0.898–0.937	0.903	0.939	0.837
OM1-OM7	0.813–0.879	0.937	0.949	0.726
NM1-NM3	0.897–0.917	0.896	0.935	0.828
CE1-CE4	0.856–0.91	0.899	0.93	0.768
EE1-EE3	0.752–0.901	0.805	0.886	0.722
AE1-AE4	0.769–0.893	0.848	0.898	0.688
EFL1-EFL11	0.68–0.852	0.937	0.946	0.615

Gen AI, General Artificial Intelligence; IM, Intrinsic Motivation; OM, Outer Motivation; NM, Amotivation; CE, Cognition of Engagement; EE, Emotion of Engagement; AE, Agentic Engagement; EFL, EFL perceived proficiency.

Discriminant validity was examined using both the Heterotrait–Monotrait ratio (HTMT) and the Fornell–Larcker criterion. All HTMT values remained below the conservative threshold of 0.90, supporting adequate conceptual distinctiveness among constructs. In addition, the square root of AVE for each construct exceeded its inter-construct correlations in most cases, confirming acceptable discriminant validity. Although several construct pairs showed relatively high correlations, these values remained below recommended thresholds and were theoretically consistent with the conceptual proximity among motivation, engagement, and proficiency.

### 3.4 Data collection and analysis procedure

All data were translated from Chinese. A pilot study was conducted to evaluate the clarity and construct validity of the instruments, and principal component analysis indicated that the items loaded strongly on their respective factors. The pilot test’s objective was to confirm the validity and dependability of the instrument to be utilized in the actual study, as well as the understanding of the statements set out in the questionnaire to reduce unanticipated problems. It enabled the researcher to redesign the research instrument if the pilot test results revealed difficulties or did not meet the required Cronbach’s alpha values at this stage.

After data screening, including the treatment of missing values, outliers, and reverse-coded items, the dataset was analyzed using SPSS 27.0 and Smart-PLS 4.0. The measurement model was evaluated by examining indicator reliability, internal consistency reliability, convergent validity, discriminant validity, and multicollinearity. Subsequently, the structural model was assessed using path coefficients, effect sizes, predictive relevance, and bootstrapping procedures. Finally, mediation analysis and PLS-Predict were conducted to evaluate indirect effects and the predictive accuracy of the proposed model.

The study followed institutional and national research ethics guidelines. Approval was obtained from the participating vocational colleges in Liaoning Province before data collection. All participants received a clear explanation of the research objectives, procedures, and their rights, and provided informed consent.

## 4 Result

### 4.1 Descriptive analysis

Data collection was conducted via a questionnaire survey. After excluding invalid responses, a total of 383 valid questionnaires were obtained. Table 2 summarizes some basic information regarding the questionnaire sample:

**Table 2:** Demographic information.

Category	Option	Frequency	Percent
Gender	Male	259	67.62%
	Female	124	32.38%
Grade	First year	308	80.42%
	Second year	28	7.31%
	Third year	47	12.27%
Major	Science	230	60.05%
	Art	83	21.67%
	Others	70	18.28%

According to survey data, males accounted for 67.62% of participants, while females constituted 32.38%. First-year students were the largest group of participants, at 80.42%. Science and engineering students were the largest disciplinary group, at 60.05%. Overall, participants represented diverse demographic backgrounds among China’s vocational students.

### 4.2 Reliability and convergent validity analysis

#### 4.2.1 Reliability analysis

The reliability and convergent validity of the measurement model were assessed by examining the indicator loadings, Cronbach’s alpha (*α*), composite reliability (CR), and average variance extracted (AVE). To ensure the reliability of the indicators, external loadings should exceed 0.70 (Hair et al., 2017).

As shown in Table 1, all measurement items demonstrated satisfactory outer loadings. Specifically, the majority of indicators loaded above the recommended threshold of 0.70, indicating strong indicator reliability despite EF10’s outer loading of 0.680 and EF11 0.697. However, if the measurement model meets the thresholds for internal consistency and convergent validity, loadings higher than 0.5 are also acceptable (Hair et al., 2017). Therefore, these values remained acceptable in exploratory and applied social science research when composite reliability and AVE meet recommended standards. Therefore, all items were retained for further analysis.

Cronbach’s alpha values ranged from 0.805 to 0.962, which exceeded the recommended minimum of 0.70. These indicated adequate to excellent internal consistency reliability across all constructs. In addition, composite reliability values ranged from 0.886 to 0.966, which confirmed the high reliability of the measurement scales. Notably, constructions such as Artificial Intelligence (AI), EFL perceived proficiency (EF), and OM demonstrated particularly strong internal consistency, with CR values well above 0.90.

Convergent validity was assessed using the AVE. All constructions exhibited AVE values exceeding the recommended threshold of 0.50 (Hu et al., 2004; Henseler et al., 2009), which ranged from 0.615 to 0.837. This indicated that each latent construct explained more than 50% of the variance in its associated indicators. These provided strong evidence of convergent validity. Constructs such as IM and NM showed especially high AVE values, which suggested a substantial shared variance among their indicators.

#### 4.2.2 Discriminant validity

A low correlation between the measured target construct and the other constructs in the research is indicative of discriminant validity (Hair et al., 2010). In Table 3, Discriminant Validity was measured by HTMT, ranging from 0.552 to 0.895. The highest HTMT association between AE and EFL perceived proficiency was 0.895, which remained within the acceptable limit, suggesting that the two constructs were empirically distinct. Their high correlation indicated conceptual relatedness, not redundancy. Motivation was typically an antecedent, while engagement functioned as a mediator linking motivation to EFL perceived proficiency. Retaining both constructs preserved the model’s explanatory power and captured the dynamic process from internal intention to observable learning behavior. Similarly, relatively high HTMT values were observed between OM and IM and between OM and CE. However, these values did not exceed the recommended cut-off, which supported adequate discriminant validity.

**Table 3:** HTMT criterion.

Variable	AE	Gen AI	CE	EE	EFL	IM	NM
AE							
Gen AI	0.552						
CE	0.759	0.763					
EE	0.787	0.652	0.832				
EFL	0.895	0.697	0.823	0.835			
IM	0.604	0.843	0.816	0.707	0.728		
NM	0.695	0.664	0.802	0.733	0.645	0.694	
OM	0.655	0.802	0.884	0.752	0.747	0.869	0.8

Gen AI, General Artificial Intelligence; IM, Intrinsic Motivation; OM, Outer Motivation; NM, Amotivation; CE, Cognition of Engagement; EE, Emotion of Engagement; AE, Agentic Engagement; EFL, EFL perceived proficiency.

The discriminant validity was also assessed using the Fornell-Larcker criterion, which compares the square root of the AVE for each construct with the correlations between constructs. As shown in Table 4, in most cases, the square root of the variance for each construct was greater than its correlation with other constructs, which indicated that the variance shared by each construct and its own indicators was greater than the variance shared with other constructs.

**Table 4:** Fornell–Larcker criterion.

Variable	AE	Gen AI	CE	EE	EFL	IM	NM	OM
AE	0.829							
Gen AI	0.508	0.818						
CE	0.668	0.713	0.876					
EE	0.651	0.587	0.718	0.85				
EFL	0.807	0.671	0.759	0.731	0.784			
IM	0.538	0.787	0.735	0.61	0.678	0.915		
NM	0.613	0.622	0.723	0.627	0.605	0.627	0.91	
OM	0.593	0.763	0.811	0.662	0.709	0.8	0.736	0.852

Gen AI, General Artificial Intelligence; IM, Intrinsic Motivation; OM, Outer Motivation; NM, Amotivation; CE, Cognition of Engagement; EE, Emotion of Engagement; AE, Agentic Engagement; EFL, EFL perceived proficiency.

Specifically, the diagonal values for AE 0.829, AI 0.818, CE 0.876, EE 0.850, IM 0.915, NM 0.910, and OM 0.852 exceeded their correlations with other constructs, which supported adequate discriminant validity. Although relatively high correlations were observed between CE and OM, AI and IM, and AE and EFL perceived proficiency, these correlations remained below the corresponding square roots of AVE. The results suggested that each construct shared more variance with its own indicators than with others. Although some correlations were relatively high, they remained below the corresponding AVE square roots, indicating acceptable conceptual distinctiveness while reflecting theoretically meaningful relationships among constructs (see Table 5).

**Table 5:** Initial eigenvalues and variance explained.

Component	Initial eigenvalue		
	Initial eigenvalue	Variance explained %	Cumulative %
1	25.402	51.841	51.841

An exploratory factor analysis including all measurement items was conducted. The unrotated factor solution revealed that the first factor accounted for 51.841% of the total variance, which was above the recommended threshold of 50% (Podsakoff et al., 2003). But acceptable. Therefore, no single factor dominated the covariance among the measurement items.

Additionally, all variance inflation factor (VIF) values ranged from 1.886 to 3.780, remaining below the conservative threshold of 5.00 (Hair et al., 2022), further suggesting that common method bias was unlikely to pose a serious threat to the validity of the findings. These results indicate that common method variance was not a substantial concern in the present study.

#### 4.2.3 Correlation analysis

The preceding analysis has confirmed that the questionnaire passed the reliability and validity tests, demonstrating good internal consistency and effective discriminability. Therefore, the collected sample data can be used for empirical analysis. To explore the relationship between Gen AI, learning motivation, learning engagement, and EFL perceived proficiency, a correlation test was conducted between the variables. The results are presented in Table 6.

**Table 6:** Pearson correlation coefficient test.

Variable	*M*	SD	1	2	3	4
1. Learning Motivation	3.791	0.752	1			
2. Learning Engagement	3.718	0.736	0.812**	1		
3. Gen AI	3.88	0.751	0.797**	0.668**	1	
4. EFL Perceived Proficiency	3.683	0.764	0.723**	0.860**	0.658**	1

*p < 0.05, **p < 0.01.

The mean scores for all four variables ranged from 3.683 to 3.880 on a Likert-type scale, indicating a moderately high level of agreement among participants. Specifically, Gen AI (*M* = 3.880, SD = 0.751) exhibited the highest mean score, followed by Learning Motivation (*M* = 3.791, SD = 0.752), Learning Engagement (*M* = 3.718, SD = 0.736), and EFL Perceived Proficiency (*M* = 3.683, SD = 0.764). The standard deviations were relatively similar across variables (ranging from 0.736 to 0.764), suggesting comparable response variability.

Pearson correlation analysis revealed several significant relationships among the variables at the *p* < 0.01 level. Learning Motivation was strongly and positively correlated with Learning Engagement (*r* = 0.812, *p* < 0.01), indicating that students with higher learning motivation tended to demonstrate greater learning engagement. Learning Motivation also showed a strong positive correlation with Gen AI (*r* = 0.797, *p* < 0.01) and a moderately strong positive correlation with EFL Perceived Proficiency (*r* = 0.723, *p* < 0.01). Learning Engagement was significantly and positively correlated with EFL (*r* = 0.860, *p* < 0.01), representing the strongest correlation in the matrix. Learning Engagement demonstrated a moderate positive correlation with Gen AI (*r* = 0.668, p < 0.01). Gen AI was significantly and positively correlated with EFL Perceived Proficiency (*r* = 0.658, *p* < 0.01), suggesting that higher levels of generative AI literacy were associated with better EFL Perceived Proficiency.

#### 4.2.4 Path analysis

**Figure 2:**
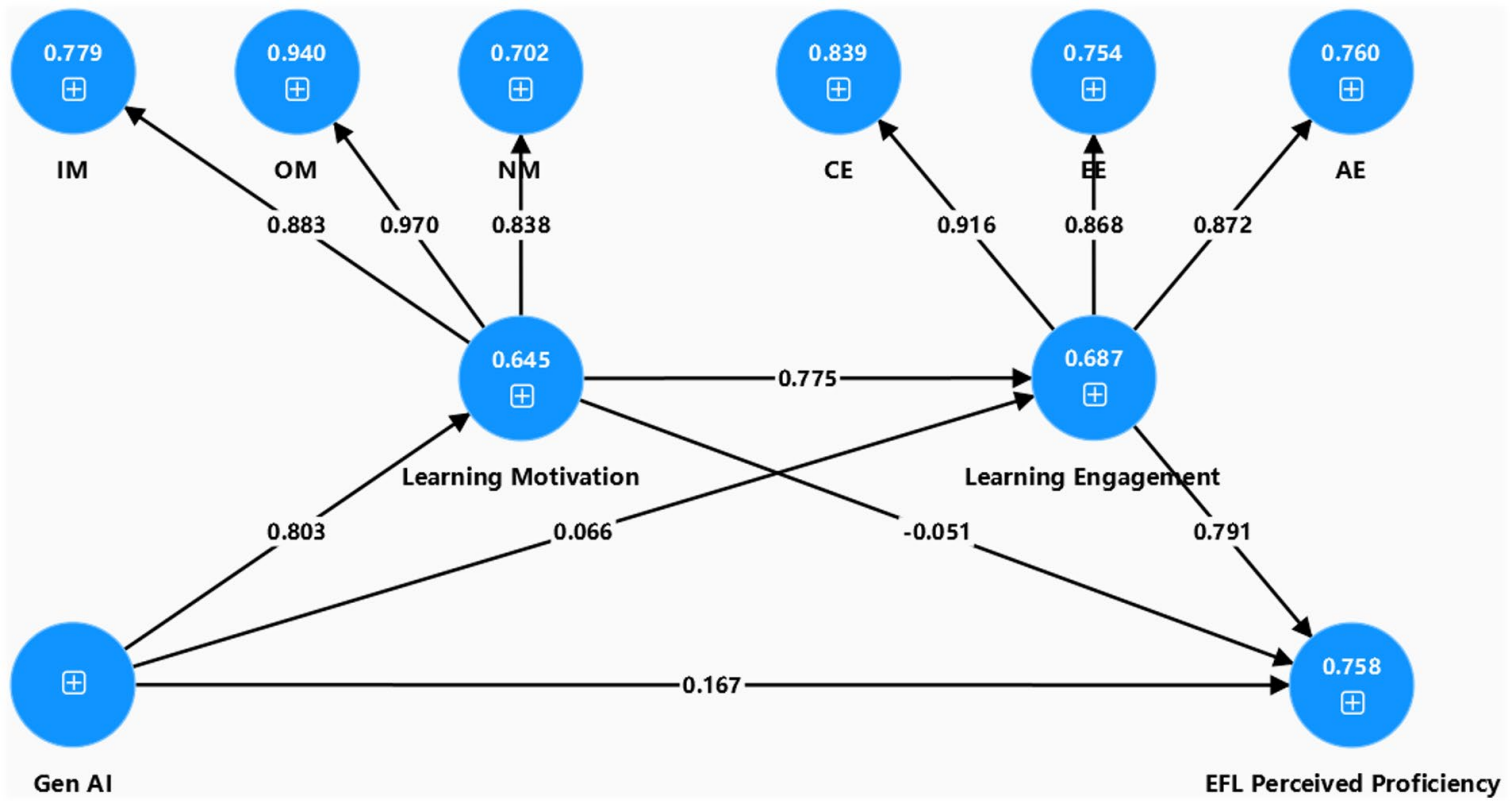
Structural path.

This study employed PLS version 4.0 to analyze and elucidate the hypothesized pathways. The specific data analysis for path testing is shown in Figure 2.

#### 4.2.5 Model fitting

The structural model demonstrated strong explanatory power. In Table 7, the R-square values indicated that the model explained 75.8% of the variance in EFL perceived proficiency. It also explained 68.7% of the variance in student engagement and 64.5% of the variance in learning motivation. According to PLS-SEM benchmarks, these values indicate strong accuracy.

**Table 7:** Predict power of *R*-squared.

Item	*R*-square	*R*-square adjusted
EFL perceived proficiency	0.758	0.756
Engagement	0.687	0.685
Learning Motivation	0.645	0.644

The adjusted R-square values were highly consistent with the original R-square values. Minimal differences between *R*^2^ and adjusted *R*^2^ suggested stable estimates without overfitting. According to the criteria proposed by Hair et al. (2019), these values represented moderate to substantial explanatory power. The high *R*^2^ values in Table 7 suggests that the model has explanatory power and can meaningfully indicate key educational outcomes in AI-supported EFL contexts. However, *R*^2^ values can only indicate the proportion of variance explained by the model, whereas the practical significance of the findings should be evaluated in conjunction with theoretical relevance, effect sizes, contextual factors, and the characteristics of the study population.

Effect sizes (*f*^2^) in Table 8 indicated differentiated predictive contributions. Gen AI showed a small effect on EFL perceived proficiency (*f*^2^ = 0.041) and a negligible effect on engagement (*f*^2^ = 0.005), and relatively large effect on learning motivation (*f*^2^ = 1.814). Engagement exerted a large effect on EFL perceived proficiency (*f*^2^ = 0.811) which should be interpreted with caution within the specific theoretical and measurement context of this study. Learning motivation had a negligible direct effect on EFL perceived proficiency (*f*^2^ = 0.002) but a large effect on engagement (*f*^2^ = 0.682), supporting its indirect influence. Table 8 showed strong practical significance, indicating key pathways to learning outcomes and highlighting engagement as a critical mechanism.

**Table 8:** Predict power of *f*-square.

Path	*f*-square
Gen AI - > EFL perceived proficiency	0.041
Gen AI - > engagement	0.005
Gen AI - > learning motivation	1.814
Engagement - > EFL perceived proficiency	0.811
Learning motivation - > EFL perceived proficiency	0.002
Learning motivation - > engagement	0.682

#### 4.2.6 Predictive correlation and collinearity VIF test

Predictive relevance was presented in Table 9. The predictive relevance of the structural model was assessed using the Stone–Geisser *Q*^2^ statistic obtained through the blindfolding procedure. *Q*^2^ values greater than zero indicate that the model has predictive relevance for a given endogenous construct (Hair et al., 2022). All endogenous constructs exhibited *Q*^2^ values greater than zero, indicating satisfactory predictive relevance.

**Table 9:** Predictive relevance (*Q*^2^) and collinearity assessment (VIF).

Construct/path	SSO	SSE	*Q*^2^ (=1-SSE/SSO)	VIF
Gen AI	5,362	2062.498	0.615	
EFL perceived proficiency	4,213	1940.363	0.539	
Engagement	4,213	2141.508	0.492	
Learning Motivation	4,979	2029.314	0.592	
Gen AI - > EFL perceived proficiency				2.828
Gen AI - > Engagement				2.814
Gen AI - > Learning Motivation				1
Engagement - > EFL perceived proficiency				3.195
Learning Motivation - > EFL perceived proficiency				4.733
Learning Motivation - > Engagement				2.814

Gen AI, General Artificial Intelligence; IM, Intrinsic Motivation; OM, Outer Motivation; NM, Amotivation; CE, Cognition of Engagement; EE, Emotion of Engagement; AE, Agentic Engagement.

The variance inflation factor (VIF) was used to test the collinearity among the predictor variables in the structural model. According to the generally accepted criteria of the PLS-SEM, a VIF value below 5.0 indicates that collinearity does not pose a serious threat to the estimation of path coefficients, while a value below 3.3 is considered an ideal level (Hair et al., 2019).

The results of the direct and indirect path analysis were also reported in Table 9. All VIFs ranged from 1.000 to 4.733, below the recommended threshold of 5.00, indicating that AI demonstrated a significant positive effect on EFL perceived proficiency, while its effect on engagement was not statistically significant. Furthermore, learning motivation was significantly associated with engagement but did not directly influence EFL perceived proficiency. The serial mediating effect of learning motivation and engagement between Gen AI and EFL perceived proficiency was found to be statistically significant.

### 4.3 Mediating effect analysis

Table 10 presents the path coefficient results. It illustrated the specific influence relationships across different pathways within the model and provided the path coefficient, T-value, and significance level (*p*-value) for each pathway.

**Table 10:** Direct and indirect path analysis results.

Path	*β*	SD	*T* statistics	*p* values	Decision
H1: Gen AI - > EFL perceived proficiency	0.167	0.052	3.235	0.001	Supported
H2: Gen AI - > Learning Motivation	0.803	0.027	29.595	0	Supported
H3: Learning Motivation - > EFL perceived proficiency	−0.051	0.088	0.582	0.561	Not Supported
H4: Learning Motivation - > Engagement	0.775	0.07	11.079	0	Supported
H5: Gen AI - > Engagement	0.066	0.071	0.936	0.349	Not supported
H6: Engagement - > EFL perceived proficiency	0.791	0.062	12.835	0	Supported
H7: Gen AI - > Learning Motivation - > EFL perceived proficiency	−0.041	0.071	0.578	0.563	Not supported
H8: Gen AI - > Engagement - > EFL perceived proficiency	0.052	0.055	0.95	0.342	Not supported
H9: Gen AI - > Learning Motivation - > Engagement - > EFL perceived proficiency	0.492	0.073	6.712	0	Supported

From the data reported in Table 10, it can be concluded that Gen AI had a significant positive direct effect on EFL perceived proficiency (*β* = 0.167, *t* = 3.235, *p* = 0.001) but did not significantly predict engagement (*β* = 0.066, *t* = 0.936, *p* = 0.349). Gen AI strongly predicted learning motivation (*β* = 0.803, *t* = 29.595, *p* < 0.001). Engagement exerted a substantial effect on EFL perceived proficiency (*β* = 0.791, *t* = 12.835, *p* < 0.001), whereas learning motivation did not directly predict EFL perceived proficiency (*β* = −0.051, *t* = 0.582, *p* = 0.561) but significantly predicted engagement (*β* = 0.775, *t* = 11.079, p < 0.001).

Mediation analysis indicated that the serial indirect effect of Gen AI on EFL perceived proficiency via learning motivation and engagement was significant (*β* = 0.492, *t* = 6.712, *p* < 0.001). However, indirect effects through learning motivation alone (*β* = −0.041, *t* = 0.578, *p* = 0.563) and engagement alone (*β* = 0.052, *t* = 0.950, *p* = 0.342) were nonsignificant. These findings supported a serial mediation pathway from Gen AI to motivation, to engagement, and ultimately to proficiency. This challenged techno-centric perspective and supported the view that learning effectiveness depends more on psychological and behavioral processes than on mere technological exposure.

## 5 Discussion

### 5.1 Gen AI is positively related to EFL perceived proficiency and learning motivation but does not directly predict engagement

The present study found that Gen AI exerted a significant positive effect on vocational students’ EFL perceived proficiency, supporting H1. This finding is consistent with previous studies indicating that AI-assisted language learning can improve language performance (Huang and Mizumoto, 2024; Law, 2024), indicating that AI-supported learning environments may facilitate language development by providing immediate feedback, personalized support, and increased access to learning resources. More importantly, Gen AI demonstrated an exceptionally strong effect on learning motivation, supporting H2. This result aligns with Self-Determination Theory (Ryan and Deci, 2000, 2020), which posits that students’ learning motivation is satisfied with the use of Gen AI. Such personalized support may be especially valuable for vocational students, who commonly report lower academic confidence and higher language anxiety than university students (Liu, 2020).

However, H5 was not supported, indicating that Gen AI did not directly predict learning engagement. This finding differs from previous studies reporting positive relationships between AI usage and engagement (Gjermeni and Prodani, 2024; Guo and Wang, 2024). One possible explanation lies in the unique characteristics of vocational education. Compared with university students, vocational learners often demonstrate fragmented learning habits, weaker self-regulation, and limited academic persistence (Liu, 2020). While Gen AI can provide learning support and increase learning convenience, vocational students may use these tools in passive or superficial ways. Another explanation may be related to the nature of AI-supported learning environments. In AI-assisted environments, learners can obtain rapid responses and task solutions with relatively little effort without producing deeper behavioral, cognitive, or emotional engagement to improve language proficiency.

### 5.2 Motivation mattered mainly through engagement rather than as an independent predictor of EFL perceived proficiency

Learning motivation did not significantly predict EFL perceived proficiency, supporting H3. This result differs from numerous studies suggesting that motivated learners generally achieve better language outcomes (Amoah and Yeboah, 2021; Ghavamnia and Kashkouli, 2022), which suggests that motivation alone cannot guarantee academic success unless it is transformed into observable learning behavior. In line with Self-Determination Theory and engagement research, motivated students may possess the desire to learn, but actual proficiency gains are more likely to occur when motivation is transformed into active engagement and consistent language-learning practice. This interpretation is further supported by the significant serial mediation pathway observed in the study.

Consistent with this interpretation, H4 was strongly supported. Learning motivation was significantly related to engagement, supporting H4. This finding is consistent with Alsmari (2023) and Liu et al. (2024a,b), who argued that motivation functions as a precursor to active learning participation, confirming that motivated vocational students are more likely to invest cognitive, emotional, and behavioral resources in learning activities.

### 5.3 Engagement has a positive relationship with EFL perceived proficiency

Among all structural relationships, engagement demonstrated the strongest direct effect on EFL perceived proficiency, supporting H6. This finding reinforces the growing consensus that engagement is the most proximal determinant of academic achievement (Hiver et al., 2021), by suggesting that engagement may serve as a critical behavioral pathway linking motivation to language-learning outcomes.

The result also partially explains why previous studies have reported inconsistent relationships between motivation and achievement. While motivation reflects learners’ internal willingness to learn, engagement reflects actual learning behavior. Language proficiency develops through repeated practice, interaction, and sustained cognitive effort rather than through intentions alone. Therefore, engagement serves as the critical mechanism through which motivational resources are converted into measurable learning outcomes. For vocational students, engagement may be particularly important because students who actively participate in classroom activities, invest cognitive effort, and regulate their own learning are more likely to overcome deficiencies in prior language preparation.

### 5.4 The serial mediation of learning motivation and learning engagement on the relationship between gen AI and EFL perceived proficiency

Neither the independent mediating effect of motivation (H7) nor the independent mediating effect of engagement (H8) was significant. These findings differ from several previous studies that reported direct mediation effects of motivation or engagement separately (Hellín et al., 2023). A possible explanation is that vocational students represent a distinctive learner population. Compared with university students, they generally exhibit greater heterogeneity in academic readiness and learning habits. Consequently, motivational enhancement alone is insufficient to produce achievement gains. Likewise, engagement cannot emerge independently without motivational support. Only when Gen AI first stimulates learners’ motivation and subsequently transforms that motivation into sustained engagement can meaningful proficiency development occur.

The most important finding of this study was the support for H9, demonstrating a significant serial mediation pathway from Gen AI to learning motivation, then to engagement, and finally to EFL perceived proficiency.

## 6 Conclusion

Overall, the findings provide a comprehensive understanding of how Gen AI supports EFL perceived proficiency among vocational college students. By identifying the serial roles of learning motivation and learning engagement, this study not only advances theoretical explanations of AI-assisted language learning but also offers practical insights for the effective integration of Gen AI in vocational English education.

### 6.1 Theoretical contributions

It extends Self-Determination Theory by positioning Gen AI as a contextual motivational affordance rather than a direct instructional determinant. Existing AI-in-education research often adopts a technology-centered perspective that treats AI exposure as the primary driver of learning outcomes. This study also clarifies the conceptual distinction between learning motivation and engagement by modeling them as serial rather than parallel constructs. Much previous research treats motivation and engagement as interchangeable predictors, which creates conceptual redundancy and weakens explanatory power. The findings show that motivation reflects internal psychological readiness, whereas engagement represents the behavioral realization of that readiness. The significant serial mediation model provides a stronger explanatory mechanism for EFL perceived proficiency improvement among vocational learners. Rather than assuming that motivation independently predicts achievement, the results show that motivation must be enacted through engagement before academic outcomes can emerge. This contributes to a more precise psychological pathway for understanding AI-supported language learning effectiveness.

### 6.2 Practical implications

For teachers, Gen AI should be integrated as a motivation-enhancing and engagement-oriented instructional tool rather than merely an efficient instrument for task completion. Classroom design should prioritize autonomy-supportive activities, reflective interaction, and cognitively demanding language tasks that require students to actively construct meaning rather than passively copy AI-generated answers.

For vocational colleges, institutional support should focus on AI literacy development rather than simple technology access. Students need explicit training in how to use Gen AI for feedback interpretation, critical thinking, and communicative practice. Without such guidance, Gen AI may increase dependency rather than improve learning quality.

### 6.3 Limitations and future research directions

The existing research on Gen AI on EFL perceived proficiency had several significant gaps. First of all, this study was conducted in Liaoning Province, China, and should be cautious to extend to the whole Chinese context. It relied on cross-sectional data collected at a single point in time, which inherently limits the ability to capture the development and dynamic interactions among variables. Future research can collect data nationwide over a longitudinal period. Secondly, causal inferences should be interpreted with caution, as the observed correlations may not fully reflect the temporal sequence or long-term effects of learning motivation, engagement, and EFL perceived proficiency. Although the observed reliability and validity indicators were high, the reliance on self-reported measurement methods may introduce common method bias. Future research should consider long-term impact of Gen AI on language learning motivation and engagement, which required a combination of qualitative methods to deepen understanding the motivational and engagement mechanisms with other potentially important mediators such as self-efficacy, academic anxiety, and teacher support. Finally, vocational students in this study represent a distinctive educational population characterized by diverse academic backgrounds and career-oriented learning goals. Future studies should compare vocational and university students across different educational contexts and cultural settings to enhance the generalizability of the findings. Such investigations would contribute to a more nuanced understanding of how Gen AI supports language learning among different learner groups.

## Data Availability

The datasets presented in this study can be found in online repositories. The names of the repository/repositories and accession number(s) can be found at: https://www.wjx.cn/newwjx/manage/myquestionnaires.aspx.

## Appendix

### Items from Gen AI Literacy questionnaire

I am able to select the appropriate AI to solve problems.

If I am curious about a suggestion made by AI, I will search for further relevant information.

I believe everyone needs AI products.

When receiving AI recommendations, I am aware that I will be responsible for acting on them.

### Items from language learning motivation scale

For the pleasure that I experience in knowing more about the literature of the second language group.

In order to get a more prestigious job later on.

Honestly, I do not know, I truly have the impression of wasting my time in studying a second language.

### Items from engagement in school-four-dimensional scale

I spend a lot of my free time looking for more information on topics discussed in class.

My school is a place where I feel integrated.

I make suggestions to teachers about how to improve classes.

### Items from Chinese perceived EFL perceived proficiency

I can understand the main content when listening to or watching radio and television programmes on general topics.

I can engage in effective oral communication or negotiation on matters of daily life, such as business, travel and shopping.

I can write short essays on topics of interest, presenting arguments and evidence, using a variety of cohesive devices to ensure semantic coherence.

I am able to understand the views, emotions, attitudes and intentions expressed by others in common social situations.
